# Numerical Investigation of Diffusioosmotic Flow in a Tapered Nanochannel

**DOI:** 10.3390/membranes12050481

**Published:** 2022-04-29

**Authors:** Sourayon Chanda, Peichun Amy Tsai

**Affiliations:** Department of Mechanical Engineering, University of Alberta, 9211 116 St. NW, Edmonton, AB T6G 1H9, Canada

**Keywords:** diffsioosmosis, tapered membrane, diffusioosmotic flow

## Abstract

Diffusioosmosis concerns ionic flow driven by a concentration difference in a charged nano-confinement and has significant applications in micro/nano-fluidics because of its nonlinear current-voltage response, thereby acting as an active electric gating. We carry out a comprehensive computation fluid dynamics simulation to investigate diffusioosmotic flow in a charged nanochannel of linearly varying height under an electrolyte concentration gradient. We analyze the effects of cone angle (α), nanochannel length (*l*) and tip diameter ($d_{t}$), concentration difference (Δc = 0–1 mM), and external flow on the diffusioosmotic velocity in a tapered nanochannel with a constant surface charge density (σ). External flow velocity (varied over five orders of magnitude) shows a negligible influence on the diffusioosmotic flow inside the tapered nanochannel. We observed that a cone angle causes diffusioosmotic flow to move towards the direction of increasing gap thickness because of stronger local electric field caused by the overlapping of electric double layers near the smaller orifice. Moreover, the magnitude of average nanoflow velocity increases with increasing |α|. Flow velocity at the nanochannel tip increases when $d_{t}$ is smaller or when *l* is greater. In addition, the magnitude of diffusioosmotic velocity increases with increasing Δc. Our numerical results demonstrate the nonlinear dependence of tapered, diffusioosmotic flow on various crucial control parameters, e.g., concentration difference, cone angle, tip diameter, and nanochannel length, whereas an insignificant relationship on flow rate in the low Peclet number regime is observed.

## 1. Introduction

Flow through miniature geometries of the order of nanometers, referred to as nanofluidics, is dominated by several key surface effects, such as electrostatic interactions, surface tension, and capillary force [1,2]. Exploiting these profound surface interactions, various fascinating micro/nano-fluidic observations, for instance, capillary condensation [3], charge selectivity [4] and DNA deformation [5], have been reported with significant applications in water purification [6,7], renewable energy [8,9], nanofabrication [10,11,12] and biosensing [13,14,15].

An intriguing electrokinetic phenomenon in nano-confinement is the motion of electrolytes along a charged surface owing to an ionic concentration gradient. This phenomenon, widely known as diffusioosmosis, is primarily governed by two essential physical mechanisms: electroosmosis (flow due to an induced electrical field within an electrical double layer) and chemioosmosis (flow due to a concentration gradient). Diffusioosmosis flow (DOF) has been studied extensively in straight 1D nanochannels for symmetric electrolytes [16,17,18,19,20,21,22,23]. A closed-form expression of one-dimensional diffusioosmotic velocity was reported by Keh et al., demonstrating its monotonic increase with increasing zeta potential [24] and non-linear increase with decreasing porosity [17,19]. Gupta et al. recently formulated a general one-dimensional analysis of diffusioosmotic velocity inside nanopores for axisymmetric electrolytes [23]. These insightful mathematical formulations were performed with 1-D Poisson–Boltzmann linearization of ion distribution and consideration of a thin electric double layer. However, the finite length of nanopores in nano/micro-junctions causes such configurations to be either quasi-2D or 3D in nature [25,26,27,28]. In this regard, numerical investigations of DOF was conducted with a 2D configuration for straight nano-confinements [29,30,31]. These 2D studies reveal an increase of diffusioosmosis flow (DOF) with increasing surface charge density [29] or increasing nanochannel aspect ratio [31], but report a nonlinear dependence of DOF on electrolyte concentration [29,30]. Researchers also reported the reversal of DOF direction at a small zeta potential of a planar charged nanochannel [19,24], at a low electrolyte concentration [29], by tuning the width-to-height ratio [31] or through specific combinations of nanochannel parameters (of *h*, *l*, and σ) [32,33].

Diffusioosmosis has been utilized for a number of engineering applications, for instance, the manipulation of micro-particles using ultraviolet lasers [34], particle motion in dead-end pores [35,36], and zeta potential measurement [37,38]. In particular, electrokinetic flow in nanochannels of various geometries, such as conical [39,40] and hourglass shapes [41,42], has been reported to demonstrate a nonlinear electrical response with low-current (i.e., high-resistance) ionic flow for a certain voltage bias, whereas a high-current (i.e., low-resistance) ionic flow occurs when the voltage bias is reversed, which is commonly referred to as ion current rectification (ICR) [43,44,45]. Due to its unique ability to promote or restrict ionic current depending on the applied voltage bias, ICR is useful and significant in several biochemical applications, such as chemical separation [46] and biomimicry [47], which in turn has promoted detailed investigations of ICR [48].

ICR in tapered nanoscale compartments has been reported to vary with EDL and tip size [49], surface charge [50], electrolyte concentration [51], and electrolyte type [52]. Inversion of ICR has also been reported due to concentration depletion at the tip [53] and at the over-limiting current threshold [54]. Research on the numerical front focused on the influence of various control parameters on ICR, for instance, the effect of nanopore tip dimension [55], non-uniform surface charge density [56,57,58], pore length [59], solution pH [60,61,62], and ionic size [63]. Concerning the tapered nanofluidics, on the one hand, numerical simulations performed by neglecting the flow field have reported surface charge density, salt concentration gradient, and nanopore geometry as the primary factors affecting ICR in tapered nanopores [48,54]. On the other hand, researchers have demonstrated the importance of electroosmotic flow in conical nanochannels by reporting significantly different ICR characteristics for systems with and without the consideration of nanoflow [64,65]. These results hence strongly imply the important consideration of the flow field for a more systematic and complete analysis in the model.

In spite of many analyses of ICR in nano-sized compartments, less attention has been given towards the detailed analysis and mechanism of the flow field through a charged, tapered nanochannel. Investigating such diffusioosmotic flow (DOF) in tapered pores can be instrumental in our understanding of selective ionic transport in biological systems [66]. In this article, we will focus on the analysis of the diffusioosmotic flow field in a tapered nanochannel under a variety of operating conditions. Through our two-dimensional numerical analysis, we aim to report the effect of several critical parameters, such as cone angle, nanochannel tip diameter and length, and concentration difference, on DOF velocity through a charged, tapered nanochannel.

## 2. Numerical Model

**Figure 1:**
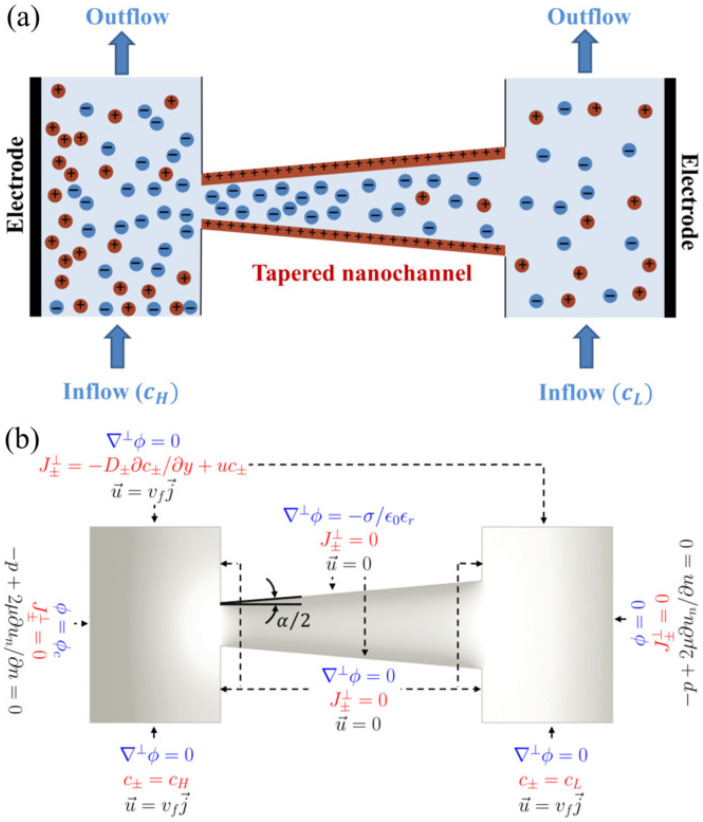
(**a**) Schematic of a positively-charged conical (diverging) nanochannel connecting two reservoirs through which electrolytes flow at a specific velocity ($v_{f}$). The geometry of tapered nanochannel is characterized by the corn angle, α. A solution at high concentration ($c_{H}$) flows through the left-hand reservoir, while a dilute solution ($c_{L}$) flows through the right-hand reservoir. The corresponding boundary conditions used in the numerical model are shown in (**b**). See Section 2 for the details of the governing equations used in the numerical model.

Figure 1a illustrates a schematic of our computational domain. In the present analysis, a two-dimensional (2D) rectangular analysis is performed where the end (or depth) effects in a tapered nanochannel are not considered. Although a complete 3D model would be more accurate for realistic nanochannels and nanopores involving multiple nanosize dimensions, our 2D analysis is appropriate for simulating a planar nanochannel system where the third dimension length scale is much greater than the nanochannel height, such as the nanochannel array system simulated with a 2D model previously [9,67,68]. In contrast, typical nanopores are cylindrical confinements where axisymmetric analysis is more accurate and should be performed.

In the current model, two reservoirs are connected with a tapered nanochannel of uniform surface charge (σ). Two solutions of different salt concentrations flow through the reservoirs ($c_{H}$ and $c_{L}$ in the left and right reservoir, respectively), generating a concentration gradient across the nanochannel. Due to the interaction between the nanochannel surface charge and ions in the electrolyte, an electric double layer (EDL) forms along the channel, which can be represented by the Debye length, $\lambda_{D}$; for a symmetric ($z_{+}=-z_{-}=z$) monovalent (z=1) electrolyte $\lambda_{D}=\sqrt{\epsilon_{0}\epsilon_{r}RT/2F^{2}c_{0}}$, where $c_{0}$ is the molar concentration of the electrolyte solution [69]. The positively charged nanochannel with overlapping EDLs repels cations and thus demonstrates charge selectivity, with an anion-exchange behavior. Due to the tapered geometry of the nanochannel, the overlapping fraction of the EDL (i.e., $\lambda_{D}/h$) will not occur uniformly throughout the length of the nanochannel. Consequently, variations in the local electric field along the nanochannel will be observed. Besides, the angle of the tapered nanochannel, α (also known as the cone angle), is expected to influence the ion concentrations and, hence, the concentration gradient along the nanochannel. Therefore, here we investigate the significant effect of α on the diffusioosmotic flow field through the nanochannel. In addition, we report the dependence of diffusioosmosis flow velocity on nanochannel length, tip diameter, concentration difference (0–1 mM), and flow rate (over five orders of magnitude). With appropriate adoptions, the present numerical model can be designed to investigate the fields of flow, ionic concentrations, and electrical potential in a micro/nano-junction, where the reservoirs can be considered to be a part of the microchannels.

### 2.1. The Governing Equations

The nanofluidic transport of ions through nano-confinements involves the coupled interactions of ions and solvent due to surface charge and local electric field, and is dictated by the conservation of mass, momentum, charge, and ionic species. Firstly, Poisson’s equation correlates the distribution of electric potential, ϕ, with the spatial distribution of net charge, *q*, due to net ionic concentrations ($c_{i}$) [70,71]:

(1) $$\begin{array}{c} \epsilon_{0}\epsilon_{r}\nabla^{2}\phi =-q=-F\sum_{i}z_{i}c_{i}, \end{array}$$

where $\epsilon_{0}\;(={8.85\times 10}^{-12}$ Fm^−1^), $\epsilon_{r}$ (= 80.1), and *F* (= 96,485 Cmol^−1^) are the permittivity of a vacuum, relative permittivity of water, and Faraday’s number, respectively. $c_{i}$ and $z_{i}$ represent the ionic concentration and valence, where the subscript *i* denotes the cation (+), or the anion (−), respectively.

Secondly, the concentration of ions, $c_{i}$ (for both $c_{+}$ and $c_{-}$), is modelled by the steady-state Nernst–Planck equation [70]:

(2) $$\begin{array}{c} \vec{\nabla }\cdot \left(-D_{i}\vec{\nabla }c_{i}-\frac{z_{i}D_{i}Fc_{i}}{RT}\vec{\nabla }\phi \right)+u\cdot \vec{\nabla }c_{i}=0, \end{array}$$

Here, $D_{i}$ is the diffusivity of the respective ion, denoted by *i* (=+,−), u is the flow velocity vector, *R* (= 8.314 J K^−1^ mol^−1^) is the universal gas constant, and *T* (= 293 K) is the temperature of the solution. In the present study, KCl is used as the dissolved salt, with $D_{+}=D_{K^{+}}=1.96\times 10^{-9}$ m${}^{2}$/s and $D_{-}=D_{Cl^{-}}=2.0\times 10^{-9}\;$m${}^{2}$/s, such that $D_{+}\approx D_{-}$. The three terms in Equation (2) represent the contributions to the ionic flux by diffusion, electromigration, and convection, respectively. Similarly, the cationic ($J_{+}$) and anionic ($J_{-}$) ion fluxes consist of the diffusive, electromigration, and convective contributions and can be obtained by integrating Equation (2) [70]:

(3) $$\begin{array}{c} J_{i}=\left(-D_{i}\vec{\nabla }c_{i}-\frac{z_{i}D_{i}Fc_{i}}{RT}\vec{\nabla }\phi \right)+uc_{i}. \end{array}$$

The current across the nanochannel cross-section, *I*, can be calculated from the integration of ionic fluxes using Equation (3):

(4) $$\begin{array}{c} I=\int F\sum_{i}\left(z_{i}J_{i}\right)\;dA. \end{array}$$

Last but not least, the steady-state Navier–Stokes equation is implemented for the investigation of velocity profile inside the nano-confinement [72]. For the present nanofluidic system, the Reynolds number, Re ($=v_{f}l/\nu$, where the nanochannel length, l∼100 nm, inflow velocity, $v_{f}\approx 10^{-6}$–$10^{-2}$ m/s, and kinematic viscosity of water, ν∼ 10${}^{-6}$ m${}^{2}$/s), varies within the range between $10^{-7}$ and $10^{-3}$. Due to such a small Re, we can neglect the contribution of the inertial terms. The velocity distribution in our geometry can, thus, be simulated using the Stokes equation, along with the Continuity equation:

(5) $$\begin{array}{c} -\vec{\nabla }p+\mu \nabla^{2}u+-q\vec{\nabla }\phi =0; \end{array}$$

(6) $$\begin{array}{cc} & \nabla \cdot u=0, \end{array}$$

where *p* is the pressure.

### 2.2. Boundary Conditions

The boundary conditions for the governing equations are represented in Figure 1b. The walls of the tapered nanochannels were considered to be positively charged, with no slip and no penetration of fluids and ions, i.e., $\vec{u}=0$ along the nanochannel surface. The positive surface charge was numerically implemented by assuming the surface charge density, σ, to be constant along the nanochannel, and can be modeled by the following equation [71]:

(7) $$\begin{array}{c} \nabla^{⊥}\phi =-\frac{\sigma }{\epsilon_{0}\epsilon_{r}}, \end{array}$$

where $\nabla^{⊥}$ symbolizes a gradient operator in the direction perpendicular to the tapered nanochannel. We investigate a steady-state flow problem by using constant and equal inflow and outflow velocities in the reservoirs. An influx of ions into the reservoirs occurred at specific concentrations, i.e., at high concentrations, $c_{H}$ (saline water), and low concentrations, $c_{L}$ (freshwater), through the left and right reservoirs, respectively. There was no external pressure difference across the nanofluidic setup. For the investigation of diffusioosmosis without any external electric field, we did not apply any external electrical potential difference in our model (i.e., we considered $\phi_{c}=0$, where $\phi_{c}$ is the electrode potential in the left-hand side).

### 2.3. Numerical Methods and Validation

Our numerical simulations were modeled using the commercial finite element-based software, COMSOL Multiphysics 5.3a. To compute our steady-state solution in the above numerical tool, we used a direct solution method, namely the multifrontal massively parallel solver (MUMPS), based on Gaussian factorization. Rigorous validation and grid independence analysis were carried out to verify our numerical model.

**Figure 2:**
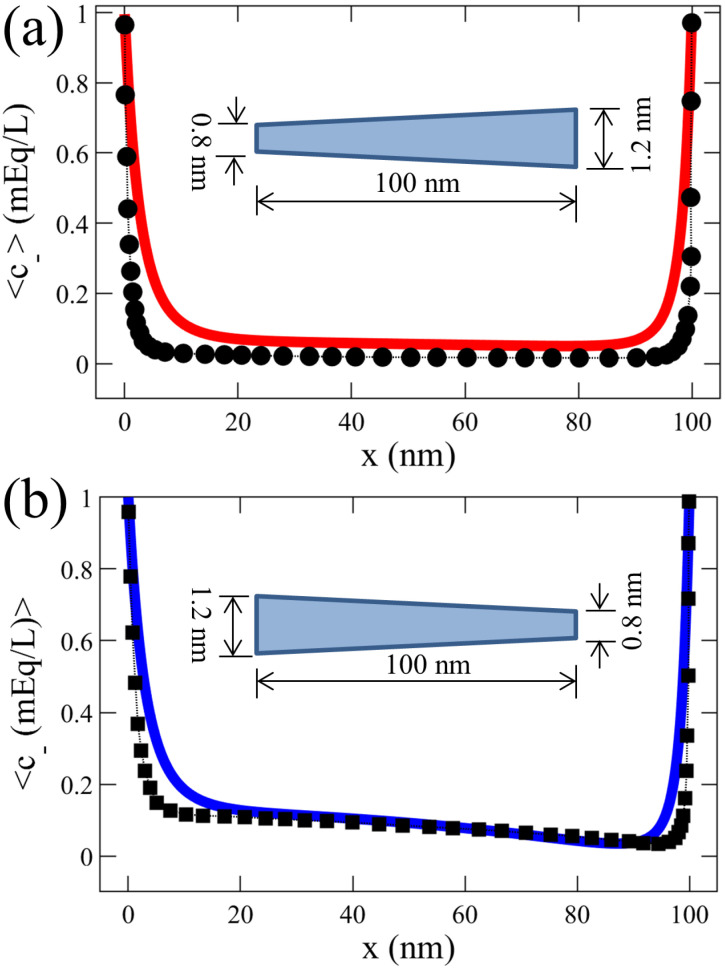
Validation of our numerical model of a tapered nanochannel by comparing the average anion concentration profiles ($c_{\pm }$) reported by Balannec et al. investigating pressure-driven ion transport through conical nanopores [73]. The geometries of the nanopores simulated are shown in the figure (not to scale), and the simulations were performed at constant surface charge density, σ=−1 mC/m${}^{2}$, and a solution concentration of 1 mEq/L (milli-Equivalent/Litre), as used by Balannec et al. The results from Balannec et al. for divergent and convergent nanopores are represented by ● in (**a**) and ◼ in (**b**), respectively. Our corresponding results are shown by — in (**a**) and — in (**b**).

Motivated by nanofiltration technologies, Balannec et al. investigated pressure-driven nanofluidic transport across conical and hourglass-shaped nanopores [73], reporting significant ICR due to co-ion exclusion at the pore tip and varying electric field along the pore. We validated our numerical model with this numerical investigation of conical nanopores [73], as shown in Figure 2. Our results of the average concentration of anions demonstrated a reasonable agreement with those reported by Balannec et al. [73]. These consistent results of insignificant $c_{-}$ values also illustrate the charge selectivity of the positively-charged nanochannel.

**Figure 3:**
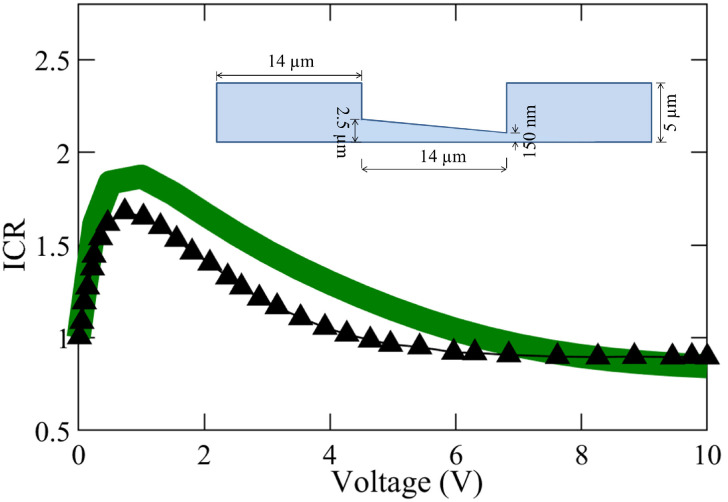
Comparison of the ion current rectification (ICR) factor from our numerical model (—) with the that from the investigation of ICR inversion effects by Rosentsvit et al. (▲) for divergent nanopores [54]. The figure inset shows the geometry (not to scale) of the axisymmetric model simulated. Following the parameters used by Rosentsvit et al., we assumed σ=−0.06 C/m${}^{2}$ and $c_{0}=1\;\mu$m for this comparison.

Shown in Figure 3 is further validation of our model performed with the results of ICR for different voltage bias, reported by Rosentsvit et al. [54]. The numerical simulations were conducted for the negative and positive voltage bias of the same magnitude, and the ICR factor was determined with the ratio of the corresponding current obtained via Equation (4) [54]:

(8) $$\begin{array}{c} \mathrm{ICR}=\frac{|I_{V_{0}<0}|}{|I_{V_{0}>0}|}, \end{array}$$

where $I_{V_{0}<0}$ and $I_{V_{0}>0}$ are the currents obtained for a negative and positive voltage bias (of the same magnitude), respectively. Overall, in spite of the complexity of the simulations and geometries, consistent values and trends of the ICR variation with voltage are obtained by our model, with our results deviating by 11.6 % from those by Rosentsvit et al. [54], where they did not consider flow in the nanochannel.

### 2.4. Mesh Independence Analysis

**Figure 4:**
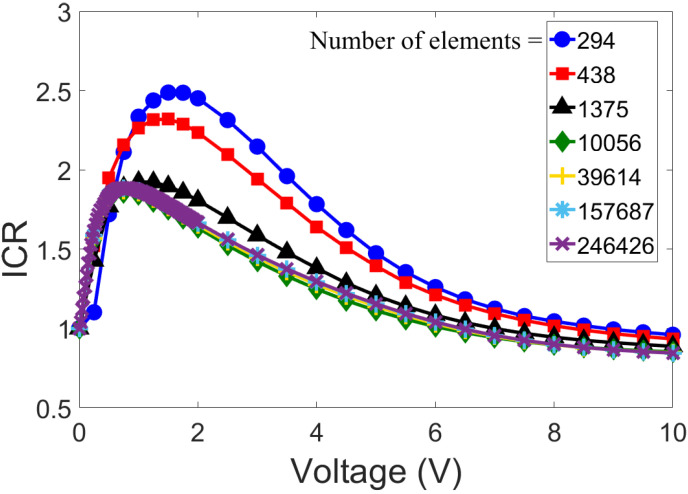
Mesh-independence studies of our numerical model, based on the ICR factor (Equation (8)) for σ=−0.06 C/m${}^{2}$ and $c_{0}=1\;\mu$m. The corresponding dimensions and model geometry are shown in Figure 3’s inset.

To perform a grid independence study, we used the same geometry as Rosentsvit et al. [54], which is described in the previous section. While keeping σ=−0.06 C/m${}^{2}$ and $c_{0}=1\;\mu$m constant, we changed the mesh size in increasing steps to identify the optimal grid spacing (with which we did not alter the simulation results). The outcomes of the mesh independence analysis are illustrated in Figure 4, with the total number of elements ranging from 294 to 246,426. With a mesh number greater than 10,000, no significant deviation of results was observed. Hence, all further numerical simulations were carried out with the total number of elements exceeding 10,000.

## 3. Results

Diffusioosmosis is primarily comprised of two essential components, namely chemioosmosis and electroosmosis. Chemioosmosis is caused due to the concentration gradient inside the nanochannel, whereas electroosmosis occurs as a result of the induced local electric field due to the EDL and surface charge [74]. In this particular case of a tapered geometry, besides these two effects, the cross-section changes along the channel length, which, based on the mass conservation, can either accelerate or decelerate the overall flow for a convergent or divergent nanochannel, respectively. A complex interplay between these effects contributes to the resultant diffusioosmosis flow through a tapered, charged nanochannel in a nonlinear manner. The geometric parameters in our model are kept more than 5 nm for all our cases, in accordance with the acceptable results for continuum modeling in nanofluidics [2].

### 3.1. Impact of Cone Angle

**Figure 5:**
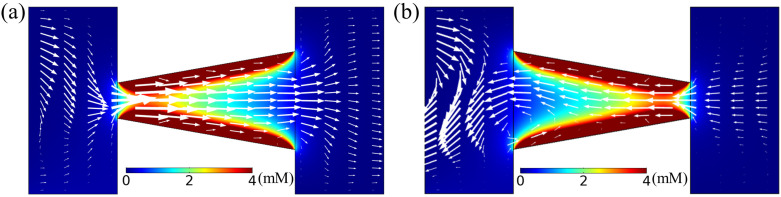
Anion concentration distribution (color map) and diffusioosmotic velocity profile (in arrows) for a nanochannel of length *l* = 100 nm and tip diameter $d_{t}$ = 20 nm for a tapered geometry of tilt angle α = 15${}^{\circ }$ (diverging) in (**a**) and α = $-15^{\circ }$ (converging) in (**b**). Here, $v_{f}$ = 7 μm/s and Δc = 0.14 mM.

We investigate the effect of cone angle (α) on diffusioosmotic flow velocity, with constant tip diameter ($d_{t}$), nanochannel length (*l*), concentration difference (Δc), and solution inflow velocity ($v_{f}$). To observe the behavior of both converging and diverging channels, we vary α in the range of ±20${}^{\circ }$, while keeping $d_{t}$, *l*, $c_{H}$, $c_{L}$ and $v_{f}$ fixed at 20 nm, 100 nm, 0.15 mM, 0.01 mM, and 7 μm/s, respectively. Typical flow fields for α = +15${}^{\circ }$ (diverging) and $-15^{\circ }$ (converging) tapered nanochannels are illustrated in Figure 5. Flow, in both cases, is directed from the edge with the smaller orifice to the wider one. Due to the effect of (positive) surface charge along the nanochannel wall, the anion concentration is higher inside the nanochannel compared to the two reservoir flows of the dilute and concentrated electrolyte solutions. There is a greater (fractional) EDL overlapping adjacent to the smaller orifice due to the narrower gap between the charged walls, as demonstrated quantitatively by the anion concentration profiles provided in the Supplementary Information. As evident from Figure 5, the concentration of anions near the smaller orifice is greater than close to the wider orifice. Therefore, a stronger concentration gradient locally develops near the smaller orifice, which results in a more dominant chemiosmotic flow (of water movement) from the nearby reservoir towards the smaller nanochannel orifice. Conversely, adjacent to the wider orifice, the concentration gradient is not as strong as in the smaller orifice. Hence, due to a smaller driving force, this flow is overtaken by the stronger chemiosmotic flow from the smaller orifice towards the wider gap.

To investigate the contribution of the electroosmotic component to these two flow scenarios, we observed the distribution of electric potential, ϕ, and electric field, E = $-\vec{\nabla }\phi$, of our simulations (with their corresponding figures given in Supplementary Materials). In both the diverging and converging nanochannels, the average electric potential at the small and large orifices was comparable, resulting in a similar electroosmotic effect. The direction of the electric field near the orifices was from the nanochannel towards the adjacent reservoirs for both diverging and converging nanochannels. Here, we noticed the maximum electric field near the center of the nanochannel, instead of close to the nanochannel tip. This is probably due to the overlapping of EDL near the nanochannel narrow tip, leading to a reduction of the electric potential gradient and, hence, the electric field. Thus, in these cases of investigations, the electroosmotic effect was found to be negligible when compared to its chemiosmotic counterpart.

**Figure 6:**
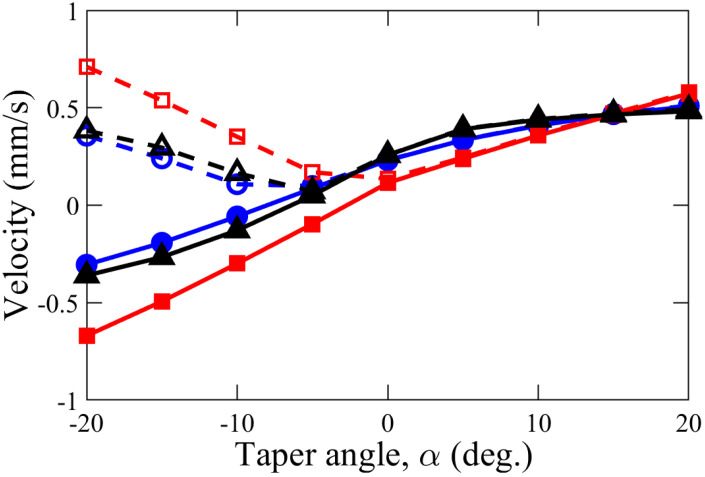
Variation of average velocity in x-direction (solid line) and velocity magnitude (dashed line) with angle of taper, α, in a nanochannel of various length *l* and tip diameter $d_{t}$: (i) *l* = 100 nm and $d_{t}$ = 20 nm (●), (ii) *l* = 100 nm, $d_{t}$ = 40 nm (◼), and (iii) *l* = 200 nm, $d_{t}$ = 20 nm (▲). Other control parameters were kept constant: Δc = 0.14 mM, $v_{f}$ = 7 μm/s, and σ = 0.01 C/m${}^{2}$.

A comparison of the diffusioosmotic flow (DOF) velocity for various taper angles, α, is shown in Figure 6 for three different scenarios of *l* and $d_{t}$. Since there is stronger DOF flow adjacent to the smaller orifice of the tapered nanochannel, the overall DOF direction is in the direction of increasing nanochannel height or gap-thickness, *h* (i.e., from a high- to low-concentration reservoir) for a positive angle of taper (α > 0). Similarly, when α is negative, DOF also occurs in the direction of increasing nanochannel height, *h* (while, in this case, directed from the low to the high concentration one). This preferable flow direction reveals that the cone angle (α) plays a vital role in the direction of DOF. In addition, the DOF flow velocity increases with an increase in the magnitude of the cone angle. When the taper angle is large, the degree of overlapping EDL along the nanochannel changes rapidly due to changing height. As a result, the electric field along the flow direction changes at a faster pace. This rapid variation in electric field due to larger cone angle causes faster movement of ionic species and, hence, higher magnitude of nanoflow velocity.

The effect of the cone angle was also investigated for different tip diameters, $d_{t}$, and nanochannel length, *l*. Keeping nanochannel length fixed (*l* = 100 nm), when the tip diameter, $d_{t}$, is changed from 20 nm to 40 nm, weaker DOF is observed when α < 15${}^{\circ }$. Moreover, in this case of $d_{t}=40$ nm, the change of DOF velocity with the taper angle was approximately linear (when compared to the other cases studied). This observation can be attributed to a substantial EDL overlap of $d_{t}$ = 20 nm, which causes a higher chemiosmotic transport, compared to a much weaker or nearly no overlap of the EDL in the case of $d_{t}$ = 40 nm. Therefore, according to the mass conservation, for a higher nanochannel $d_{t}$ without overlapping of the EDL, the diffusioosmotic velocity in the ’2D’ geometry changes in an inversely linear fashion with the change in flow cross-sectional area ($A=d_{t}\cdot w$, where *w* is the thickness of the nanochannel and can be assumed to be of unity for the 2D geometry). Keeping $d_{t}$ = 20 nm, when *l* was changed from 100 nm to 200 nm, an insignificant variation in the DOF results were observed.

### 3.2. Effect of Tip Diameter and Nanochannel Length

**Figure 7:**
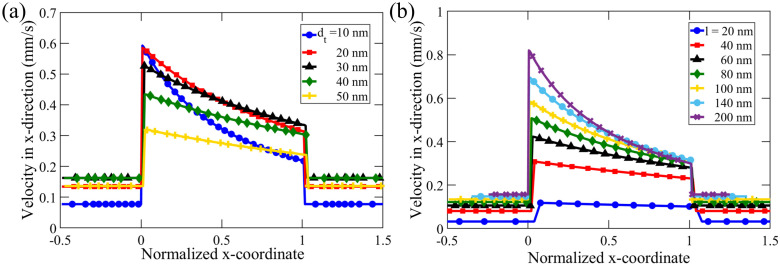
Average velocity in the x-direction along a nanochannel of constant cone angle, $\alpha =10^{\circ }$, inflow velocity, $v_{f}=7\;\mu$m/s, and concentration difference, Δc=0.14 mM to study the effect of (**a**) nanochannel tip diameter, $d_{t}$, in the range 10–50 nm, with *l* = 100 nm, and (**b**) nanochannel length, *l*, in the range 20–200 nm, with $d_{t}=20$ nm. The x-coordinate is normalized with the nanochannel length, *l*.

To carefully analyze the effect of nanochannel length, *l*, and tip diameter, $t_{p}$, we analyzed the x-directional velocity profile inside the nanochannel, illustrated by Figure 7. To facilitate better visualization, we non-dimensionalized the x-coordinate along the nanochannel with the nanochannel length, *l*, with the zero (reference point) in the normalized x-coordinate representing the left-hand side edge of the tapered nanochannel. Under a constant cone angle, nanochannel length, concentration difference, and inflow velocity, we observed the effect of varying $d_{t}$ on DOF in the range of 10–50 nm, as shown in Figure 7a. In all the simulations, we observed the maximum flow velocity at the narrowest orifice of the nanochannel. This observation is consistent with the mass conservation principle because the flow rate remains constant throughout the nanochannel, and, hence, the maximum velocity would occur near the minimum flow cross-section area.

Theoretically, DOF velocity decreases with the increase in the diameter along the tapered nanochannel based on mass conservation. Even though the taper angle and nanochannel length are the same for all the simulations, we observe that the decrease in velocity with increasing channel height is steeper for a smaller tip diameter. With α and *l* kept constant for all the simulations in Figure 7a, we observe that the diffusioosmotic velocity decreases as the gap-diameter increases, with the maximum value at the (narrow) tip (with corresponding diameter and cross-sectional area represented by $d_{t}$ and $A_{t}$, respectively) and the minimum amount at the (broad) base of the tapered nanochannel (with the corresponding diameter and area denoted as $d_{b}$ and $A_{b}$ respectively). This decrease in DOF is more rapid for nanochannels with a smaller $d_{t}$. For instance, in case of $d_{t}=$ 10 nm, the flow velocity near the base ($v_{b}$) is about half of that at the tip ($v_{t}$), i.e., $v_{t}/v_{b}\approx 2$, which is consistent with the corresponding (cross-sectional) area ratio $A_{t}/A_{b}=d_{t}/d_{b}\approx 1.88$). In addition, for $d_{t}=$ 50 nm, average DOF velocity is comparable at the tip and the base; in this case, $v_{t}/v_{b}\approx 1.2$ is consistent with the ratio of cross-sectional area, $A_{t}/A_{b}\approx 1.35$. This suggests that the effect of the tip diameter is enhanced for smaller $d_{t}$-values, when the EDL overlap is more pronounced, which in turn causes a stronger concentration gradient.

DOF decreases non-linearly for smaller $d_{t}$ (10–20 nm). The overlapping of the EDL can cause higher concentrations in the nanochannel, and, hence, results in a higher concentration difference when compared to the electrolyte concentration in the reservoir. This results in a stronger chemiosmotic effect, contributing to an overall higher diffusioosmotic flow for smaller $d_{t}$. However, in the case of larger $d_{t}$ (30–50 nm), there is a much lesser EDL overlap near the smaller orifice (See SI for quantitative analysis). The above effect of higher DOF due to EDL overlap was not observed in channels with wider tips. The flow velocity in nanochannels with larger $d_{t}$, thus, shows an approximately linear decrease from the tip to the base along the length of the nanochannel due to dilation of the 2D cross-sectional area, which is proportional to the gap thickness. For further illustration, we looked at the simulation results shown in Figure 7a, and found that a tapered nanochannel with $d_{t}$ = 50 nm has a much smaller average anion concentration ($\langle c_{-}\rangle$) of 0.65 mM at the tip when compared to that of a nanochannel with $d_{t}$ = 20 nm ($\langle c_{-}\rangle =$1.33 mM). As a result, the corresponding diffusioosmotic velocity near the nanochannel tip is u = 0.32 mm/s in case of $d_{t}$ = 50 nm, which is much less than for $d_{t}=20$ nm (*u* = 0.58 mm/s).

To obtain a complete picture of the effect of dimensions for tapered nano-confinements, we vary the nanochannel length (*l*) while keeping the other parameters constant. Figure 7b shows the results of our investigation of variation of *l* in the range of 20–200 nm. The maximum speed increases with an increase in *l*, even when $d_{t}$ remains constant. With a larger *l*, the total surface charge is higher, and thus, the overall effect on DOF is stronger via the electroosmotic component of the DOF, which is manifested by a higher DOF velocity at the nanochannel tip. The minimum speed inside the nanochannel is of comparable magnitude for various nanochannel lengths for l>20 nm. Figure 7b further demonstrates that the decreasing rate of the flow velocity (in the normalized x-coordinate) is nonlinear. The rate is higher near the narrow end of the nanochannel. Moreover, this decrease in velocity is more pronounced for a longer nanochannel (i.e., for larger *l*). This nonlinearity in the velocity field might arise due to the fractional overlap of EDL. Overlap of EDL occurs when the nanochannel height (*h*) is comparable to the Debye length [32]. However, since *h* changes along a tapered nanochannel, a fractional overlap of EDL is encountered, which gives rise to nonlinearity in flow dynamics (see the Supplementary Material for more explanation).

### 3.3. Influence of Concentration Difference

**Figure 8:**
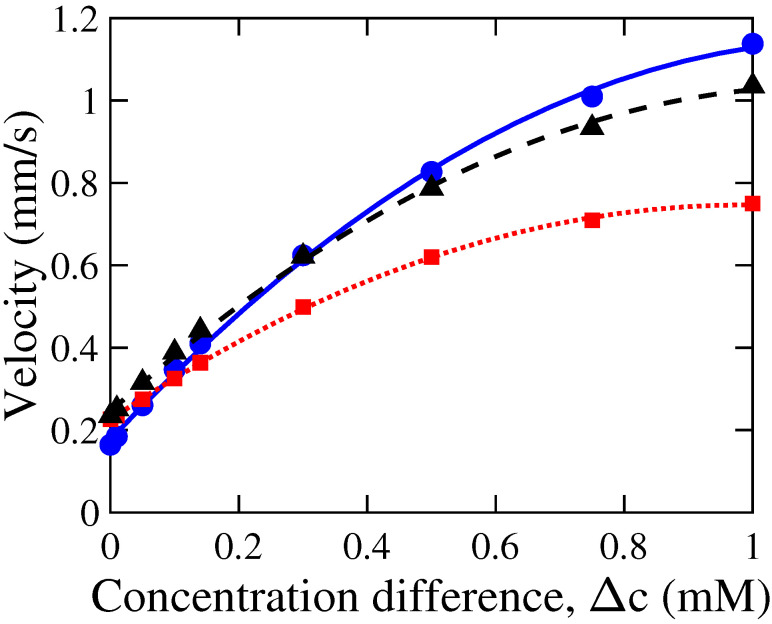
Variation in the magnitude of the average velocity, $|<u_{DO}>|$, with concentration difference, Δc, in a nanochannel. Simulation results are shown for different parameters of nanochannel length, *l*, and tip diameter, $d_{t}$: (i) *l* = 100 nm, $d_{t}$ = 20 nm (●), (ii) *l* = 100, $d_{t}$ = 40 nm (◼), and (iii) *l* = 200, $d_{t}$ = 20 nm (▲). The data points represent our simulation results, whereas the corresponding lines were obtained by a quadratic fit of these data points. Other control parameters were kept constant: α = 10${}^{\circ }$, $v_{f}$ = 7 μm/s, and σ = 0.01 C/m${}^{2}$.

We investigated the effect of concentration difference on the DOF velocity in a tapered nanoduct by varying the concentration $c_{H}$ while keeping $c_{L}$ constant at 0.01 mM. The external concentration difference, $\Delta c\;(=c_{H}-c_{L})$, was varied from 0 to 1 mM while maintaining constant values of the other control parameters, as illustrated by Figure 8. The diffusioosmotic velocity shows a nonlinear increase with an increase in Δc for all the three simulated cases of different nanochannel dimensions. The increase in DOF velocity with Δc empirically shows an excellent fitting of a quadratic polynomial of the form of $u_{DO}\approx a{\left(\Delta c\right)}^{2}+b\Delta c+c$, where *a*, *b* and *c* are fitting results (and may be a function of the nanochannel parameters). The quadratic fitting results for all three geometries under investigation are shown in Figure 8. For example, in case of a tapered nanochannel of $d_{t}$ = 20 nm, *l* = 100 nm, and $\alpha =10^{\circ }$, the fitting results show a=0.75, b=1.7, and c=0.17. This quadratic relation observed for the tapered nanochannel is in contrast to a linear dependence of DOF velocity with Δc observed for a straight, charged nanochannel [9].

The nonlinearity may arise due to the changing gap thickness, which causes the fractional EDL overlapping (i.e., λ/h) along the nanochannel to vary. This partial overlap of the EDL causes variation in local (anion) concentration along the channel, affecting the chemioosmotic flow along the channel. Furthermore, the electric field, perturbed by the local variations in concentration, influences the electroosmotic component. The complex interplay between chemioosmotic and electroosmotic flows depends on Δc, contributing to a nonlinear dependence of DOF velocity on Δc. When the tip diameter was increased from 20 nm to 40 nm, a significant reduction in DOF magnitude was observed. This decrease in DOF flow is caused by the decreasing fraction of the overlapping EDL from the case of *h* = 20 nm to 40 nm, as $\lambda_{D}\approx$ 10 nm on a single-charged wall when $c_{0}\approx$ 1 mM. For instance, in case of Δc=1 mM, the average velocity inside the tapered nanochannel decreased from 1.15 mm/s for $d_{t}$ = 20 nm to 0.75 mm/s for $d_{t}$ = 40 nm. Moreover, the nonlinearity in Δc dependence also increases. These observations may be due to the fact that the nanochannel tip diameter, $d_{t}$, affects the overlap of the EDL, and hence, the overall flow. On the other hand, an increase in nanochannel length from 100 nm to 200 nm, while keeping $d_{t}$ constant at 20 nm, did not cause a noticeable variation in the DOF velocity for different Δc in the range of 0–1 mM.

Even when there was no concentration difference across the tapered nanochannel (i.e., Δc=0), non-zero DOF was still observed inside the tapered nanochannel. This flow can probably be attributed to the taper in the nanochannel, resulting in local concentration differences along the channel due to partial EDL overlap. This local concentration difference and induced electric field drive localized chemiosmotic and electroosmotic effects in tapered nanochannels, causing an overall flow.

### 3.4. Effect of External Flow

**Figure 9:**
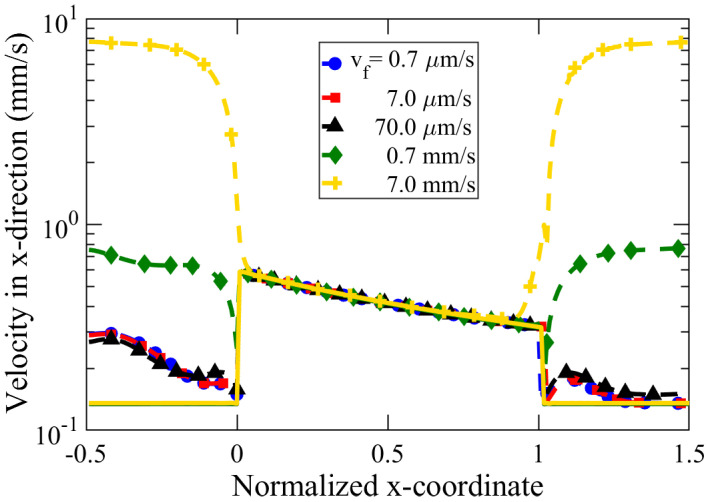
Average velocity in the x-direction (solid lines) and velocity magnitude (dashed lines) along a nanochannel of length l=100 nm, tip diameter, $d_{t}=20$ nm, cone angle, $\alpha =10^{\circ }$, and concentration difference, Δc=0.14 mM. The x-coordinate has been normalized with the nanochannel length, *l*.

We studied the effect of inflow velocity, varying over five orders of magnitude in the range of 0.7 μm/s–7 mm/s. The inflow velocity, $v_{f}$, was kept equal for both the left (high concentration, $c_{H}$) and right (low concentration, $c_{L}$) reservoirs. Figure 9 reveals our simulation results of the diffusioosmotic flow velocity for various $v_{f}$ in a semi-logarithmic plot. For the range of flow velocities analyzed, the inflow velocities did not alter the flow pattern inside the nanochannel. A higher inflow velocity (e.g., $v_{f}$= 7 mm/s) causes an increase in the y-component of DOF near the edges of the tapered nanochannel, illustrated in Figure 9 by the high value of the velocity magnitude. However, we observe the insignificant impact of the inflow rate on the DOF speed inside the tapered nanochannel, as the velocity in the x-direction and velocity magnitude matched with each other. Hence, the flow inside the nanochannel is dominated by x-directional flow, with an insignificant y-directional velocity component. Such an observation is likely due to our investigation in the low Peclet number regime ($Pe=v_{f}h/D≲0.1$, where $v_{f}\leq$ 7 mm/s, h≤ 50 nm, $D≃2\times 10^{-9}$ m${}^{2}$/s). Here, higher flow velocity was not investigated, since such high flow velocities are difficult to implement and are rarely reported for micro/nano-fluidic experiments. In case of very high inflow velocity ($v_{f}=$ 7 mm/s) in Figure 9, we observed that a large reservoir inflow velocity could influence the DOF near the edges of the tapered nanochannel, which is manifested in terms of high-velocity magnitude near the nanochannel orifices.

## 4. Conclusions

We present a two-dimensional numerical analysis of diffusioosmosis in nanochannels of tapered geometry to investigate the importance of various operating parameters, such as cone angle, nanochannel dimensions, and flow rate. In contrast to previous research, which focuses mainly on the ICR response of tapered nano-confinements, we investigate the flow dynamics inside the tapered nanochannel and how it is affected by various control parameters. Understanding the nanoflow inside the nano-confinements is crucial for achieving precision in the design of micro/nano-fluidic systems and applications involving tapered nano-confinements.

In the low Peclet number regime (Pe≲0.1), an insignificant variation of DOF inside the nanochannel was observed when the external flow velocity of solutions through the reservoirs was varied over five orders of magnitude. The angle of taper was found to have a very prominent effect on the direction of diffusioosmotic velocity, where a positive taper angle (i.e., diverging shape) facilitates flow in the direction towards increasing gap thickness (i.e., from a high- to low-concentration reservoir in our setup). In contrast, a negative taper angle favors flow in the opposite direction from low to high concentration (but still in the direction towards increasing gap thickness). The primary reason for such an observation is likely due to the overlap of EDL at the narrower orifice, causing a higher concentration gradient, which in turn promotes greater chemiosmotic flow (from the nearby reservoir towards the smaller orifice via water transport). By the same token, a smaller tip diameter increases the DOF.

Due to the influence of the surface charge on the ionic concentration distribution inside the nanochannel, a longer nanochannel length increases the flow velocity via a higher net surface charge and, hence, electroosmotic effect. In contrast to a previous analysis in straight and charged nanochannels reporting a linear dependence of DOF velocity on Δc [9], a nonlinear increase in DOF with an increase in concentration difference was observed, which can be modeled via an empirical relation of a second-order polynomial. The nonlinearity of this dependence increases when the tip diameter is smaller, whereas a change in the channel length did not cause a significant impact on the overall DOF.

In brief, this investigation reports that the diffusioosmotic flow in a tapered nano-confinement depends nonlinearly on concentration difference, cone angle, tip diameter, and nanochannel length. Our simulation results demonstrate a significant diffusioosmotic flow field in a tapered charged nanochannel under Δc and, therefore, the importance of flow modeling in relevant diffusioosmosis applications as well as nanofluidic platforms and technologies. The present research was performed in a two-dimensional numerical arrangement of a charged, tapered nanochannel. Future work can be extended to a 3-D model of diffisioosmotic flow in a nanopore, providing insights into flow dynamics and electrical responses in a variety of 3D realistic nano-confinements.

## Data Availability

Data available upon reasonable request.

## Supplementary Materials

The following supporting information can be downloaded at: https://www.mdpi.com/article/10.3390/membranes12050481/s1, Figure S1: Electric potential distribution (color map) and electiric field (in arrows), for a nanochannel of *l* = 100 nm, $d_{t}$ = 20 nm, and α = (a) 15${}^{\circ }$, and (b)-15${}^{\circ }$, with $V_{f}$ = 7 µm/s and *▵c* = 0.14 mM. References [69,75] are cited in the supplementary materials.
