# Supplementary material for: Numerical Investigation of Diffusioosmotic Flow in a Tapered Nanochannel
Source: Membranes (Basel). 2022 Apr 29;12(5):481. doi: 10.3390/membranes12050481 (PMC9143036; doi:10.3390/membranes12050481)
Supplement: Supplementary file 1 [file membranes-12-00481-s001.zip › membranes-1673590-supplementary.pdf]

(Dated: April 29, 2022)

Abstract

In this supplementary material, firstly, we provide quantitative details about the possible overlap of electric double layer (EDL) near the narrower orifice. We provide a quantitative comparison of the velocity ratio from our numerical simulations with 1D analytical predictions. Lastly, we also provide an investigation of electroosmotic effect through plots of electric potential distribution and electric field in the model geometry.

OVERLAPPING OF EDL

To quantitatively explain our observations, we perform an approximate analysis based on the average anion concentration at the nanochannel orifices. For the cases shown in Fig. 5 of the main manuscript, d_t=20 nm, α=±10°, l=100 nm, $d_b = 37.63$ nm, $c_H = 0.15$ mM and $c_L = 0.01$ mM. Average anion concentration at the base of the nanochannel, $c_{-,b} \sim 1$ mM and, thus, for z=1, the Debye length, λ_D (= $\sqrt{\epsilon_0 \epsilon_r RT/2F^2 c_0}$ [1]) ≈ 9.2 nm. Given that $d_b = 37.63$ nm, $d_b \nsim 2\lambda_D$. Similarly, average anion concentration at the nanochannel tip, $c_{-,t} \sim 4/3$ mM, and, thus, $\lambda_D \approx 8$ nm. For $d_t = 20$ nm, $d_t \sim 2\lambda_D$, and there is a much greater possibility of EDL overlap in this case.

DIFFUSIOOSMOTIC VELOCITY AT TIP AND BASE OF THE NANOCHANNEL

Due to constant surface charge density, σ, we assume the effect of electroosmotic component to be similar in both the tip and base of the nanochannel (which is quantitatively explained in the following section), and the difference in flow is due to the chemioosmotic component, u_{CO}. Thus, ignoring any contribution due to electroosmosis, we assume $u_{DO} \sim u_{CO}$, and compare our results with 1D analytical predictions of u_{DO} through scaling analysis [2]. In the two simulation cases under investigation, we vary the angle of taper, and keep all other control parameters fixed. Thus, the contribution of most variable towards diffusioosmosis can be expected to be constant, and thus, we can assume $u_{DO} \sim u_{CO} \propto \Delta c/L$. Now, since $c_{-,t}$ (or $c_{-,b}$) $\gg c_H$ (or c_L), for a simplified analysis, we assume $\Delta c \sim c_{-,t}$ for the nanochannel tip, and $\Delta c \sim c_{-,b}$ for the nanochannel base. Also the length scale, L, is assumed to be equal to d_t or d_b in the smaller and larger orifice respectively. With these assumptions, we have:

$$\frac{u_{DO,t}}{u_{DO,b}} \approx \frac{c_{-,t}/d_t}{c_{-,b}/d_b} = \frac{4d_b}{3d_t} \approx 2.5 \tag{S1}$$

To compare the 1D analytical results with out 2D simulations, we only consider the velocity in x-direction (i.e. $u_{DO} \approx u_x$). From our simulation for α=+10°, we, thus, have $u_{DO,t} \approx 0.6$ mm/s, and $u_{DO,b} \approx 0.3$ mm/s. Thus, $u_{DO,t}/u_{DO,b} \approx 2$, which agrees with the analytical predictions with 25% deviation. This does prove that analytical 1D predictions are a great starting point for qualitative comparison. However, the large deviation points out that detailed numerical simulations is necessary for quantative accuracy.

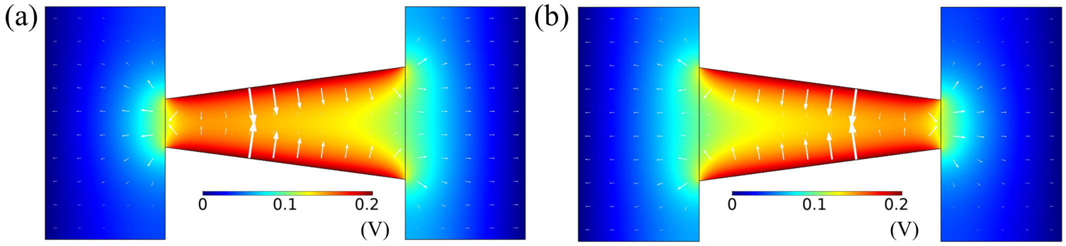

FIG. S1 Electric potential distribution (color map) and electiric field (in arrows), for a nanochannel of l=100nm, d_t=20 nm, and α=(a) 15°, and (b)-15°, with V_f=7 μm/s and Δc= 0.14 mM.

ELECTRIC POTENTIAL DISTRIBUTION AND ELECTRIC FIELD IN THE NANOCHANNEL

The distribution of electric potential, ϕ and electric field, $\mathbf{E}$=(-$\vec{\nabla}\phi$) for two simulation cases of different cone angle, α = ±15° are shown in Fig. S1 . In both the diverging and converging nanochannels, the average electric potential at the small and large orifices were similar. This would likely result in comparable electroosmotic effect near the orifices. For both diverging and converging nanochannels, the electric field is always directed away from the nanochannel towards the reservoirs. Interestingly, electric field was found to be maximum near the centre of the nanochannel, and not near the nanochannel tip. This observation can probably be attributed to to the overlapping of EDL near the nanochannel tip. EDL overlap combines the exponentially varying electric potential profiles from the charged nanochannel surfaces on either boundaries, which results in a higher combined electric potential. At the same time, this results in a reduction of the electric potential gradient, and hence the electric field. Smaller electric field near the orifices results in smaller electroosmosis, and hence, the diffusioosmotic flow in tapered nanochannels with fractional EDL overlap is dominated by chemioosmosis, will a smaller contribution from its electroosmotic counterpart.

[1] R. J. Hunter, *Foundations of Colloid Science*, Oxford University Press, 2nd edn., 2001.

[2] D. C. Prieve, *Adv. Colloid Interface Sci.*, 1982, **16**, 321–335.
